# Lymphangitis in a Portuguese Patient Infected with *Rickettsia sibirica*

**DOI:** 10.3201/eid1403.070680

**Published:** 2008-03

**Authors:** Rita de Sousa, Luís Duque, José Poças, Jorge Torgal, Fátima Bacellar, Juan P. Olano, David H. Walker

**Affiliations:** * National Institute of Health, Lisbon, Portugal; † Hospital de São Bernardo, Setubal, Portugal; ‡ Universidade Nova de Lisboa, Lisbon, Portugal; § University of Texas Medical Branch, Galveston, Texas, USA

**Keywords:** Rickettsia, spotted fever group, rickettsia sibirica strain mongolotimonae, Rhipicephalus pusillus, Portugal, lymphangitis, letter

**To the Editor**: We report a case of *Rickettsia sibirica* mongolitimoniae strain infection associated with lymphangitis (*1*). A 44-year-old man was admitted to São Bernardo Hospital in Setubal, Portugal, on August 21, 2006. Twelve days previously while on vacation at Troia Peninsula, he noted malaise, insomnia, and dry buccal mucosa. The next day he observed a small erythematous pruritic lesion on the lower right forearm that 2 days later developed into an eschar. He also had fever and sought medical care. After treatment with topical bacitracin, floxacillin, and acetaminophen for 2 days, fever (38.7°C) continued with lymphangitis extending from the right wrist to the elbow. The medication was changed to nimesulide. Three days later a rash developed on the trunk and arms, and lymphangitis extended to the axilla. Fever and chills continued, leading to hospital admission. No history of tick exposure was reported. Physical examination showed blood pressure 128/73 mm Hg, pulse 96/min, and a rubbery, nontender right supraclavicular lymph node ≈1 cm in diameter. Several 5- to 10-mm maculopapular erythematous lesions were observed on the patient’s palms. He had inflammation on the right forearm suggestive of lymphangitis and an eschar with surrounding edema and erythema on the dorsal lower right forearm (Figure). Admission evaluation showed platelets 117,000/μL, total bilirubin 0.42 mg/dL, albumin 3.42 g/dL, creatinine 1.1 mg/dL, alanine aminotransferase 244 U/L, aspartate aminotransferase 54 U/L, alkaline phosphatase 1061 U/L, creatine phosphokinase 87 U/dL, lactate dehydrogenase 784 U/L, C-reactive protein 7.1 mg/dL, radiographic pulmonary diffuse reticular pattern, arterial pO_2_ 68 mm Hg, O_2_ saturation 94%, pCO_2_ 22 mm Hg, and arterial blood pH 7.35. The differential diagnoses included rickettsiosis, pneumonia, and cellulitis. Treatment with vancomycin, cefatriaxone, and 100 mg of doxycycline twice a day was begun. On the day after hospitalization, a heparinized blood sample and 2 skin biopsy samples were collected. Vancomycin and ceftriaxone were discontinued at 48 hours when rickettsial infection was confirmed by PCR on skin biopsy; 48 hours later, the patient was afebrile.

**Figure:**
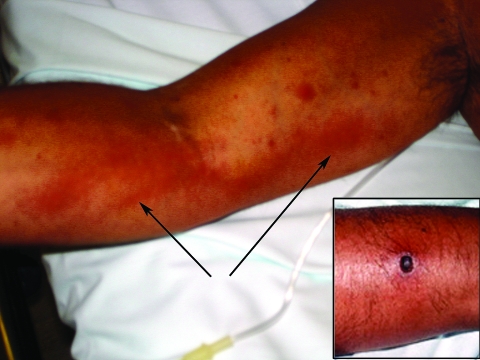
Lymphangitis extending from the right forearm to the axilla and (inset) eschar on right forearm, caused by *Rickettsia sibirica* mongolitimonae strain. Arrows indicate lymphangitis.

Immunofluorescence assay for antibodies that used *R. sibirica* mongolitimonae strain as antigen demonstrated seroconversion with no antibodies in the serum sample collected on August 21 and immunoglobulin G (IgG) and IgM antibodies at a titer of 256 in serum collected on August 30. DNA was extracted from 1 skin biopsy sample by using a DNeasy Tissue Kit (QIAGEN, Hilden, Germany). The products of nested PCR showed 100% similarity with *gltA* (353/353) and *ompA* (350/350) nucleotide sequences of *R. sibirica* mongolitimonae strain (GenBank accession nos. DQ423368.1 and DQ423367.1) (*1*).

Cutaneous biopsy indicated epidermal and dermal necrosis with extensive lymphocyte- and macrophage-rich inflammatory infiltrates involving the papillary and reticular dermal blood vessels characteristic of rickettsial infection. Relatively scant intracellular organisms were observed in the reticular dermis by spotted fever group rickettsia–specific immunohistochemistry (*2*).

Because of the presence of shared protein and lipopolysaccharide antigens in spotted fever group rickettsiae, distinguishing infections with closely related rickettsiae such as *R. conorii, R. africae*, and *R. sibirica* by serologic or immunohistochemical methods is very difficult. However, isolation and/or PCR detection followed by genetic characterization can determine the genotype of the organism to the level of genus, species, and strain. The incidence of *R. sibirica* mongolitimonae strain infection in Portugal is not known because the usual laboratory confirmation by serologic methods does not distinguish these cases from Mediterranean spotted fever.

Both cases of *R. sibirica* infection that have been recognized in Portugal occurred in August during the seasonal peak of Mediterranean spotted fever (*1*). This epidemiology differs from that in other countries (*3*–*6*). Perhaps differences in seasonal activity, population dynamics, or species of the vectors are the basis for the varying epidemiology. In Portugal, *R. sibirica* has been detected in *Rhipicephalus pusillus* (*1*). *Rickettsia sibirica* mongolitimonae strain was first isolated from a *Hyalomma asiaticum* tick from Inner Mongolia in 1991 (*7*) and subsequently from *H. truncatum* in Niger and from *H. excavatum* removed from a Greek patient (*4*,*8*).

Lymphangitis in some patients with *R. sibirica* mongolitimonae strain infection is a potentially useful diagnostic sign. Nevertheless, half of the patients with reported cases have not had lymphangitis, and infections caused by other *Rickettsia* spp. can also cause lymphangitis (e.g., patients with African tick bite fever and *R. heilongjiangensis* infections) (*1*,*3*–*6*,*9*,*10*). Thus, the diagnosis of any rickettsiosis should not be based solely on clinical manifestations. The pathogenic role of rickettsiae in lymphangitis remains to be determined. *R. sibirica* may possibly infect the endothelium of the lymphatic vessel along the pathway from the rickettsial portal of entry at the eschar inoculation site to regional lymph nodes. However, rickettsiae have not been observed in lymphatic vessels, and the lymphatic vessel lesion has not been characterized. The possibility of another agent or pathogenic effectors cannot be excluded. Currently, this clinical manifestation is the strongest evidence that rickettsiae may initially spread by a lymphogenous route before hematogenous dissemination.
